# Predicting feed intake in confined beef cows

**DOI:** 10.1093/tas/txae001

**Published:** 2024-01-04

**Authors:** Megan A Gross, Amanda L Holder, Alexi N Moehlenpah, Harvey C Freetly, Carla L Goad, Paul A Beck, Eric A DeVuyst, David L Lalman

**Affiliations:** Department of Animal and Food Sciences, Oklahoma State University, Stillwater, OK 74078, USA; Division of Agriculture and Natural Sciences, College of the Ozarks, Branson, MO 65726, USA; Department of Animal and Food Sciences, Oklahoma State University, Stillwater, OK 74078, USA; USDA, ARS, U.S. Meat Animal Research Center, Clay Center, NE 68933, USA; Department of Statistics, Oklahoma State University, Stillwater, OK 74078, USA; Department of Animal and Food Sciences, Oklahoma State University, Stillwater, OK 74078, USA; Department of Agricultural Economics, Oklahoma State University, Stillwater, OK 74078, USA; Department of Animal and Food Sciences, Oklahoma State University, Stillwater, OK 74078, USA

**Keywords:** beef cow, dry matter intake, prediction equations

## Abstract

Six existing equations (three for nonlactating and three for lactating; NRC, 1987, Predicting feed intake of food-producing animals. Washington, DC: The National Academies Press, National Academy of Science; doi: 10.17226/950; NRC, 1996, Nutrient requirements of beef cattle, 7th Revised Edition: Update 1996. Washington, DC: The National Academies Press; doi: 10.17226/9791; Hibberd and Thrift, 1992. Supplementation of forage-based diets. J. Anim. Sci. 70:181. [Abstr]) were evaluated for predicting feed intake in beef cows. Each of the previously published equations are sensitive to cow-shrunk BW and feed energy concentration. Adjustments in feed intake prediction are provided for level of milk yield in NRC (1987. Predicting feed intake of food-producing animals. Washington, DC: The National Academies Press, National Academy of Science; doi: 10.17226/950) and NRC (1996 Nutrient requirements of beef cattle, 7th Revised Edition: Update 1996. Washington, DC: The National Academies Press; doi: 10.17226/9791) equations. The equation published in 1996 used data generated between 1979 and 1993. Our objectives were to validate the accuracy of the published equations using more recent data and to propose alternative prediction models. Criteria for inclusion in the evaluation dataset included projects conducted or published since 2002, direct measurement of feed intake, adequate protein supply, and pen feeding (no metabolism crate data). After removing outliers, the dataset included 53 treatment means for nonlactating cows and 32 treatment means for lactating cows. Means for the nonlactating dataset were dry matter intake (DMI) = 13.2 ± 2.9 kg/d, shrunk body weight (SBW) = 578 ± 83.9 kg, body condition score = 5.7 ± 0.73, and Mcal net energy for maintenance (NEm)/kg of feed = 1.27 ± 0.15 Mcal/kg. Means for the lactating dataset were DMI = 14.6 ± 2.24 kg/d, SBW = 503 ± 73.4 kg, body condition score = 4.7 ± 0.58, and Mcal NE_m_/kg feed = 1.22 ± 0.16. Simple linear regression was used to determine slope, intercept, and bias when observed DMI (*y*) was regressed against predicted DMI (*x*). The NRC (1996. Nutrient requirements of beef cattle, 7th Revised Edition: Update 1996. Washington, DC: The National Academies Press; doi: 10.17226/9791) nonlactating equation underestimated feed intake in diets moderate to high in energy density with intercept differing from 0 and slope differing from one (*P* ≤ 0.01). Average deviation from observed values was 2.4 kg/d. Similarly, when the NRC (1996. Nutrient requirements of beef cattle, 7th Revised Edition: Update 1996. Washington, DC: The National Academies Press; doi: 10.17226/9791) equation was used to predict DMI in lactating cows, the slope differed from one (*P *< 0.01) with average deviation from observed values of 3.0 kg/d. New models were developed by pooling the two datasets and including a categorical variable for stage of production (0 = nonlactating and 1 = lactating). Continuous variables included study-average SBW^0.75^ and diet NE_m_, Mcal/kg. The best-fit empirical model accounted for 68% of the variation in daily feed intake with standard error of the estimate Sy root mean squared error = 1.31. The proposed equation needs to be validated with independent data.

## Teaser

An independent dataset was used to evaluate equations available to estimate feed intake in beef cows. One equation (Hibberd and Thrift, 1992. Supplementation of forage-based diets. J. Anim. Sci. 70:181. [Abstr]) provided a reasonably accurate estimate of feed intake for lactating cows, although the equation from the same authors overestimated feed intake in nonlactating cows.

## Introduction

An accurate estimate of feed intake is a fundamental component necessary to determine nutrient balance and project animal performance (Fox et al., 1995). In the beef cattle industry, large commercial feed yards, receiving yards and research institutions measure, monitor, and manage feed intake of growing and finishing cattle routinely. From these datasets, empirical models were developed and validated for the purpose of predicting feed intake of growing and finishing cattle (NRC, 1984, 1987, 1996; Anele et al., 2014; NASEM, 2016). Comparatively, little data are available to develop, validate, and refine empirical models intended to predict feed intake in beef cows (NRC, 1987; Galyean and Gunter, 2016; Lalman et al., 2019). Extensive, non-confined management systems that predominate beef cow production in the United System limit direct feed intake measurement to research institutions and confinement housing conditions.

The National Academy of Sciences, Engineering, and Medicine (NASEM) beef cattle committee has published several equations intended to provide general guidance for the feed intake of beef cows (NRC, 1984, 1987, 1996; NASEM, 2016). These equations included a considerable amount of feed intake data calculated from internal or external marker-based approaches. However, Neal et al. (1984) suggested that prediction equations using data from marker-based intake estimates were inferior to datasets containing direct measurements of intake along with relevant characteristics of animals and forage. One influential component in the most recent and widely used equation for beef cows (NRC, 1996; NASEM, 2016) is an adjustment for milk yield in lactating cows. This model component was adapted from dairy cow data (NRC, 1987) and has not been validated for beef cows. The objective of this work was to evaluate beef cow feed intake prediction equations using more recent data limited to direct measurement approaches.

## Materials and Methods

### Data Screening

A literature search and screening process was conducted for recent beef cow forage or feed intake data. Published and unpublished data were identified through *Journal of Animal Science*, *Translational Animal Science*, *Applied Animal Science*, PubMed, Google Scholar, personal communication, and recent datasets from Oklahoma State University’s Range Cow Research Center. The first screening criteria imposed included only data based on voluntary, ad libitum feed intake management. The most recent beef cow intake equation recommended by NRC (1996) and NASEM (2016) was developed using experimental data collected from 1979 to 1993. Therefore, to avoid datasets used in that analysis, the second search criteria restricted inclusion to projects conducted or published between 2003 and 2022. A second objective for restricting inclusion to more recent studies was to capture potential long-term genetic and management changes that might influence feed intake in beef cows.

Third, only direct measurements of feed intake data were included. Challenges associated with marker-based feed intake data for grazing animals were recently reviewed by Galyean and Gunter (2016). Included in these challenges (potential sources of error) are relatively small numbers of experimental units per treatment mean, brief intake measurement periods, accurate determination of grazed diet nutritive value, potential for inconsistent marker dosing, and/or incomplete marker recovery (Langlands et al., 1974; Cordova et al., 1978; Holechek et al., 1982). Coleman et al. (2014) suggested that alkanes may overestimate digestibility and therefore magnify intake estimates. While direct intake measurements overcome some of these problems, pen-based intake estimates suffer from some of the same challenges. For example, there is a plethora of published data employing a 10- to 14-d adaptation period followed by 5- to 7-d of direct feed intake measurement. To improve accuracy for individual animal intake estimates within contemporary groups, the Beef Improvement Federation recommends 42 d of feed intake data collection to ensure a minimum of 35 d of reliable measurements. Additionally, feed intake determined in pen-fed animals does not reflect additional energy required for grazing activity and other behavioral differences (Coleman et al., 2014). We excluded studies using metabolism crate housing.

Fourth, only data from experiments identified as having provided adequate protein supply to meet ruminal and animal requirements were included. There were no screening criteria applied to diet forage or concentrate proportions. Diets included in the dataset ranged from 35% to 100% forage (DM basis). Finally, studies reporting feed intake of lactating cows were required to include an estimate of milk yield.

After applying the selection criteria, available datasets predominantly utilized *Bos taurus* cattle with British or British/Continental breed influence. Data sources, number of means used from each experiment, and general classification for stage of production are provided in Table 1.

**Table 1.** Summary of sources for beef cow feed intake data

First author, year	Source^1^	No. of treatment means	Stage of production
Andresen et al. (2020)	J	2	Gestation
Banta et al. (2008)	J	5	Gestation
Briggs et al. (2022)	J	1	Gestation
Cassaday (2016)	T	4	Gestation
Freetly et al., personal communication	U	3	Gestation
Freetly et al. (2020)	J	1	Nonpregnant/nonlactating
Gross et al. (2020)	T	1	Gestation
Holder et al., unpublished data	U	2	Gestation
Holder et al., unpublished data	U	2	Gestation
Holder et al. (2019)	T	2	Gestation
Holder et al. (2022)	J	2	Gestation
Holder et al. (2020)	T	2	Gestation
Holder et al. (2022)	J	2	Gestation
Jardstedt et al. (2018)	J	2	Nonpregnant/nonlactating
Johnson et al. (2003)	J	4	Gestation
Martin et al. (2019)	J	1	Gestation
Moehlenpah et al. (2021)	J	5	Nonpregnant/nonlactating
Moore et al. (2022)	T	1	Gestation
Mourer (2012)	T	2	Gestation
Sexten et al. (2021)	J	4	Gestation
Walker et al. (2015)	J	2	Gestation
Warren (2017)	T	3	Gestation
Black et al. (2013)	J	3	Lactation
Cassaday et al. (2016)	T	2	Lactation
Gross et al., unpublished data	U	1	Lactation
Gross et al. (2020)	T	1	Lactation
Holder et al. (2019)	T	1	Lactation
Holder et al. (2021)	T	2	Lactation
Johnson et al. (2003)	J	4	Lactation
Johnson et al. (2003)	J	4	Lactation
Moore et al. (2022)	T	1	Lactation
Mourer et al. (2012)	T	2	Lactation
Mourer (2012)	T	2	Lactation
Walker et al. (2015)	J	2	Lactation
Williams et al. (2018)	T	4	Lactation
Winterholler et al. (2009)	J	3	Lactation

^1^Source: J, journal; T, thesis, abstract, or research report; U, unpublished data.

The qualitative data included average trial cow-shrunk body weight (**SBW**), study-average body condition score when available (**BCS**; Wagner et al., 1988), dry matter intake (**DMI**), diet net energy for maintenance (**NE**_**m**_, Mcal/kg), supplement DMI, supplement NE_m_, and milk yield when applicable. Insufficient information was available to determine study-average days pregnant or days in milk in several experiments. Therefore, stage of production was limited to two classification variables: nonlactating or lactating. Unless SBW was described and reported directly, cow BW was converted to SBW by multiplying BW by 0.96 (NASEM, 2016). Reported diet NE_m_ values were used when available. In cases where NE_m_ was not reported, NE_m_ was calculated from diet composition according to ingredient tabular values (NASEM, 2016). Treatment or period mean, standard deviation (**SD**), minimum and maximum values for both stages of production are shown in Table 2. Where supplement was provided, the contribution of supplement to daily DMI and NE_m_ was included. Therefore, observed daily DMI and NE_m_ intake represent the sum of contributions from the basal diet plus supplement.

**Table 2.** Mean, standard deviation, minimum, and maximum for observed variables

Item	Number of treatment means	Mean	STD	Min	Max
Nonlactating	53				
Cow SBW, kg^a^		589	76.7	420	730
BCS^b^		5.8	0.75	4.4	7.5
DMI, kg^c^		12.9	2.90	8.3	20.6
Diet NE_m_, Mcal/kg^d^		1.25	0.16	0.93	1.54
Lactating	32				
Cow SBW, kg^a^		510	69.3	404	692
BCS^b^		4.8	0.58	4.1	6.9
DMI, kg^c^		14.4	2.1	10.3	19.2
Diet NE_m_, Mcal/kg^d^		1.21	0.15	0.99	1.49
Daily milk yield, kg		6.56	2.3	3.0	11.3

^a^Study-average shrunk body weight, kg.

^b^Body condition score, scale = 1 to 9 (Wagner et al., 1988).

^c^Feed intake, kg/d (dry matter basis).

^d^Diet net energy for maintenance, Mcal/kg.

### Calculations and Statistical Analysis

A total of 98 (60 nonlactating and 38 lactating) treatment means met the screening criteria. Of the 60 nonlactating observations, nine represented cows that were described as nonpregnant, with the remaining classified as pregnant. Within stage of production, observations were further evaluated for outliers. Outliers were determined using residuals calculated by regressing observed DMI on predicted DMI with diet NE_m_, SBW^0.75^, and stage of production in the regression model. Outliers were defined as observations with studentized residuals greater than three (in absolute value; SAS Inst. Inc., Cary, NC).

Three prediction equations for gestating cows and three prediction equations for lactating cows were evaluated: NRC (1987), Eq. A; NRC (1996), Eq. B; and Hibberd and Thrift (1992), Eq C and D (Table 3). The Hibberd and Thrift (1992) feed intake guidelines for beef cows were first presented in tabular form and have been used for many years in extension and popular press publications. These guidelines were approximated in graphical form in the NASEM (2016) publication and subsequently, regression equations were developed using the original tabular values (T.A. Thrift, personal communication, September 2018). Resulting equations are shown in the footnotes for Table 3.

**Table 3.** Parameter estimates for regression of observed feed intake on predicted feed intake (kg DM/d) for gestating and lactating beef cows

Stage	Equation	*r* ^2^	RMSD^a^	Intercept^b^	Slope^c^
Gestation	Equation A (NRC, 1987)^d^	0.56	3.1	−1.84 ± 1.8	*1.40 ± 0.17*
	Equation B (NRC, 1996)^e^	0.46	2.4	*−5.33 ± 2.7*	*1.52 ± 0.23*
	Equation C (Hibberd and Thrift et al., 1992)^f^	0.47	2.8	0.35 ± 1.9	*0.85 ± 0.13*
Lactation	Equation D (NRC, 1987)^g^	0.75	4.1	1.89 ± 1.1	*1.19 ± 0.11*
	Equation E (NRC, 1996)^h^	0.66	3.0	−1.5 ± 2.2	*1.36 ± 0.18*
	Equation F (Hibberd and Thrift et al., 1992)^i^	0.67	1.5	*4.85 ± 1.2*	*0.68 ± 0.09*

^a^RMSD = root mean squared deviation expressed as kg/d.

^b^Intercept ± SE. italic font indicates intercept values significantly different from 0 at *P* < 0.05.

^c^Linear regression coefficient ± SE. Italic font indicates coefficients significantly different from 1 at *P* < 0.05.

^d^Equation A: DMI, kg/ *d* = SBW^0.75^, kg * (0.0194 + 0.0545 * NE_m,_ Mcal/kg).

^e^Equation B: DMI, kg/ *d* = (SBW^0.75^, kg * [0.04997 * NE_m_^2^ + 0.04631])/ NE_m_, Mcal/kg.

^f^Equation C: DMI, kg/ *d* = (−0.0323 * NE_m_^2^, Mcal/kg + 0.0944 * NE_m_, Mcal/kg—0.0418) * SBW, kg.

^g^Equation D: DMI, kg/ *d* = SBW^0.75^, kg * (0.0194 + 0.0545 * NE_m,_ Mcal/kg) + 0.2 * milk yield, kg/d.

^h^Equation E: DMI, kg/ *d* = ([SBW^0.75^, kg * [0.04997 * NE_m_^2^ + 0.04631] + 0.2 * milk yield, kg/d])/ NE_m_, Mcal/kg.

^i^Equation F: DMI, kg/ *d* = (−0.0261 * NEm^2^ + 0.07777 * NEm—0.0277) * SBW, kg.

Evaluation for each of the six equations was performed using the PROC REG procedure in SAS (v. 9.4; SAS Inst. Inc., 2013). Observed DMI values (Y; kg/d) were regressed against predicted DMI values (X; kg/d) according to (Piñeiro et al. 2008)


$$\begin{array}{l} Y=a+bXi+Ei\; \end{array}$$ (1)

An *F*-test (*P <* 0.05) was used to determine null hypotheses intercept (*a*) = 0 and slope (*b*) = 1. If the slope is statistically different from 1 (null hypothesis is rejected), the predicted values are not consistently related to observed values. Similarly, if the slope is not different from 1 but the intercept is statistically different from 0, then the model is biased, consistently under- or overestimating daily DMI. If both null hypotheses are accepted (*P* *>* 0.05), then disagreement between observed and predicted values are due to unexplained variance (Piñeiro et al., 2008).

The coefficient of determination for simple linear regression (*r*^2^) was calculated as an indication of the proportion of the linear variation of observed values (*y*) explained by the variation of predicted values (*x*). Root mean squared deviation (RMSD) was calculated to determine the deviation of predicted values against the *y* = *x* or unity line expressed in the same units as the model variable (kg/d; Kobayashi and Salam, 2000; Gauch et al., 2003; Piñeiro et al., 2008).

Two new feed intake prediction models were developed using the evaluation dataset. The first approach was like that employed by NRC (1996), where a prediction equation was produced to estimate daily kcal NE_m_ intake/ kg SBW^0.75^ (NEMI). Total NE_m_ intake was determined as the product of DMI and dietary NE_m_, Mcal/kg. Candidate predictor variables included diet NE_m_, $N{E_{m}}^{2}$, a class variable for stage of production (STAGE; 0 = nonlactating, 1 = lactating), and the interaction of NE_m_ by STAGE. Stage of gestation (first, second, or third trimester) and stage of lactation (early or late) were not included because of lack of uniformity in timing for feed intake measurement within STAGE. Similarly, milk yield was not included as a predictor variable because of the lack of uniformity among experimental protocols in terms of timing (early, mid, or late lactation) and milk yield measurement procedures. Predicted daily NEMI was then divided by diet NE_m_ to estimate daily feed intake (kg/d). In the second approach, a prediction equation was developed to estimate feed DMI (kg/d) directly. Predictor variables included SBW^0.75^, diet NE_m_, diet $N{E_{m}}^{2}$, STAGE, NE_m_ by STAGE, and SBW^0.75^ by STAGE. Coefficient of determination (*r*^2^) and standard error of the estimate (Sy.x) were used to determine goodness of fit for the new models.

The CALIS procedure of SAS (v. 9.4; SAS Inst. Inc., 2013) was used to develop standardized path coefficients between components in the models. Path coefficient probabilities were calculated using a *t*-test. Diet NE_m_ and STAGE were exogenous variables meaning their variance was not determined by other variables in the model. DMI and SBW^0.75^ were treated as endogenous variables indicating their variance was determined by other variables in the model.

## Results and Discussion

### Data Screening

In one experiment, feed intake was measured for nonlactating, nonpregnant cows first consuming grass hay, and then later consuming corn silage (Martin et al., 2019). The mean for corn silage intake was removed from the dataset because feed intake of the corn silage was unreasonably low and met the criteria for exclusion as an outlier. In addition, six treatment means for gestating and six treatment means for lactating cows consuming a pelleted straw/alfalfa hay or pelleted alfalfa hay diet, respectively, met the exclusion criteria due to exceptionally high feed intake (Parsons et al., 2021). These modifications resulted in the availability of 53 observations for nonlactating cows with a range in diet NE_m_ of 0.93 to 1.54 Mcal/kg and 32 observations for lactating cows with a range in diet NE_m_ of 0.99 to 1.49 Mcal/kg.

### Evaluation of Existing Equations for Nonlactating Beef Cows

**Figure 1.**
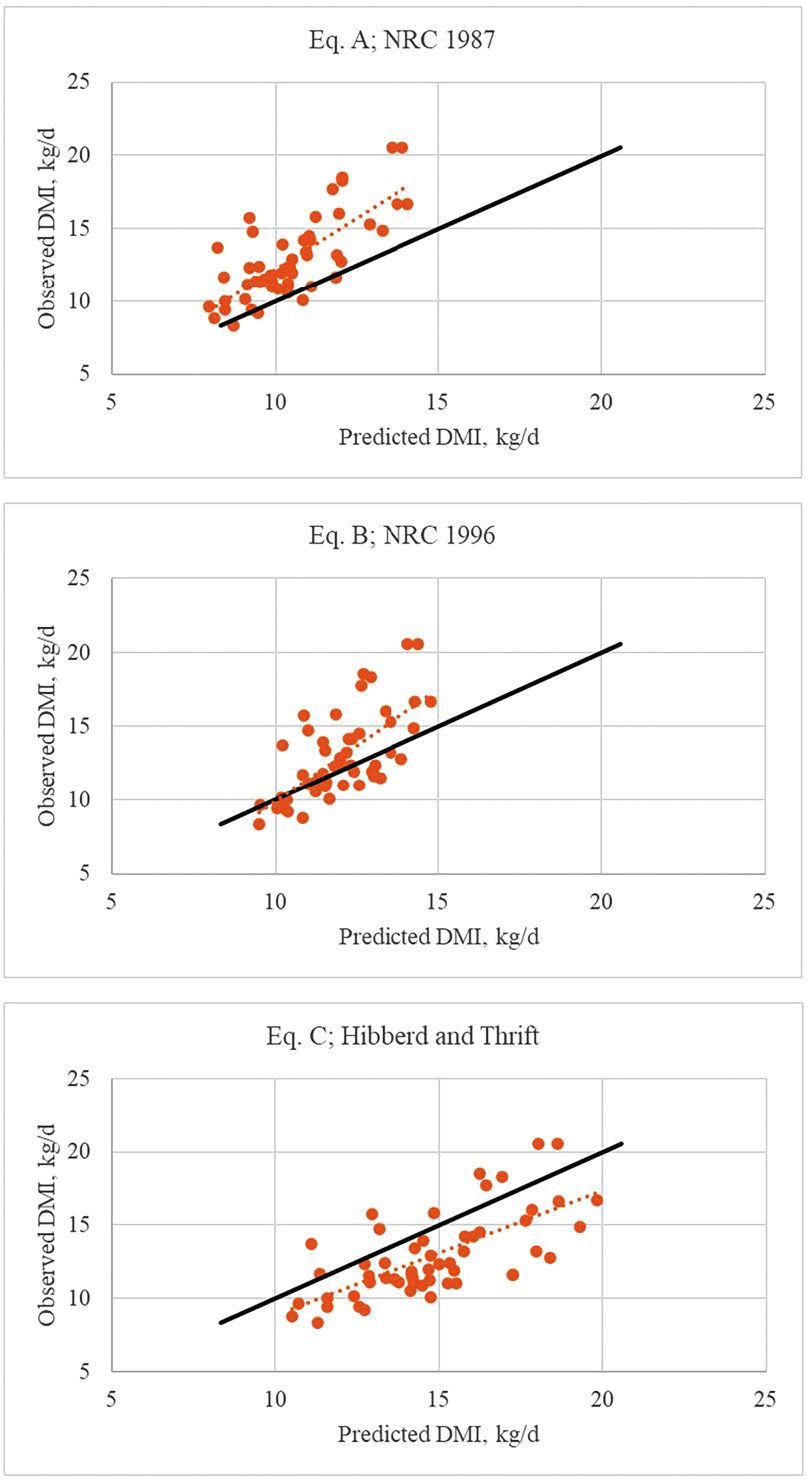
Relationship of observed to predicted feed intake in nonlactating cows using equation A (NRC, 1987), equation B (NRC, 1996), and equation C (Hibberd and Thrift, 1992).

Results from regressing predicted feed intake for nonlactating cows against observed feed intake values are shown in Figure 1 and Table 3. The intercept differed (*P =* 0.05) from 0 for equation B, while the slope differed (*P <* 0.01) from 1 for all three equations. Interestingly, all three equations provided reasonable estimates of feed intake at the lower range of diet energy concentration. Equations A and B underestimated feed intake while equation C overestimated feed intake with increasing diet energy density. While predictions from equation A explained more of the variation in observed values compared to equations B and C, the average error of prediction ranged from 2.4 to 3.1 kg/d (RMSD), suggesting lack of fit for all three equations. The fit statistic, RMSD, should not be confused with standard error of the estimate (Sy.x), which in this case is the same statistic as root mean squared error (RMSE) because there is only one parameter in the regression model. RMSD is an estimate of the average error between observed values and the unity line or *y* = *x*. In contrast, RMSE is a measure of the average error or deviation between observed values and the line derived from regressing *y* on *x*. Therefore, RMSD can only equal RMSE if the regression is perfect (intercept = 0 and slope = 1.0). Otherwise, RMSD will be larger than RMSE. Therefore, RMSD calculated by regressing observed (*y*) against predicted (*x*) values, provides a better evaluation of model performance when prediction is the objective (Piñeiro et al., 2008). For example, even though the RMSE (1.9, data not shown) for equation A, is lower than equations B and C (2.1, data not shown), equation A produced inaccurate predictions of forage intake with average error of 3.1 kg/d.

Prediction error for equations A and C can be attributed to a combination of bias and unexplained variance (Table 4). In contrast, a large percentage of equation B prediction error was from unexplained variance.

**Table 4.** Squared sum of prediction error and Theil’s partial inequality coefficients for regression of observed on predicted feed intake (kg DM/d) in gestating and lactating beef cows

Stage	Prediction equation	SSPE^a^	U_bias,_ %^b^	U_slope,_ %^b^	U_error,_ %^b^
Gestation	Equation A (NRC, 1987)^c^	515	59	4	37
	Equation B (NRC, 1996)^d^	302	15	8	77
	Equation C (Hibberd and Thrift et al., 1992)^e^	416	43	2	55
Lactation	Equation D (NRC, 1987)^f^	534	95	1	4
	Equation E (NRC, 1996)^g^	277	79	3	18
	Equation F (Hibberd and Thrift et al., 1992)^h^	69	6	22	72

^a^SSPE,  squared sum of prediction error.

^b^Theil’s coefficients reflecting proportion of variance of observed values not explained by the predicted values (SSPE) partitioned into *U*_bias_, the proportion associated with mean differences between observed and predicted values, *U*_slope,_ the proportion associated with the slope of the fitted model and the *y* = *x* line, and *U*_error,_ the proportion associated with the unexplained variance.

^c^Equation A: DMI, kg/ *d* = SBW^0.75^, kg * (0.0194 + 0.0545 * NE_m,_ Mcal/kg).

^d^Equation B: DMI, kg/ *d* = (SBW^0.75^, kg * [0.04997 * NE_m_^2^ + 0.04631])/ NE_m_, Mcal/kg.

^e^Equation C: DMI, kg/ *d* = (−0.0323 * NE_m_^2^, Mcal/kg + 0.0944 * NEm, Mcal/kg—0.0418) * SBW, kg.

^f^Equation D: DMI, kg/ *d* = SBW^0.75^, kg * (0.0194 + 0.0545 * NE_m,_ Mcal/kg) + 0.2 * milk yield, kg/d.

^g^Equation E: DMI, kg/ *d* = ([SBW^0.75^, kg * [0.04997 * NE_m_^2^ + 0.04631] + 0.2 * milk yield, kg/d])/ NE_m_, Mcal/kg.

^h^Equation F: DMI, kg/ *d* = (−0.0261 * NEm^2^ + 0.07777 * NEm—0.0277) * SBW, kg.

Equation A was developed using the data of Vona et al. (1984). In that experiment, mature, nonlactating beef cows were fed long-stemmed warm-season grass hays harvested at different stages of maturity. This dataset has several unique characteristics rarely found in the literature. First, 35 different hay lots were fed over 2 yr with a wide range in NEm (0.76 to 1.78 Mcal/kg; NRC, 1996). Secondly, forage intake and fecal output were measured directly, resulting in a relatively large dataset employing in vivo forage intake and apparent digestibility methods. Nevertheless, several factors could contribute to the substantial underprediction of the more recent data using the equation derived from this classical dataset. Fifteen of the thirty-five hay lots contained <8% crude protein (DM basis) with nine lots containing between 4.8% and 7.5% crude protein (DM basis; Vona et al., 1984). It is well established that feed intake and diet digestibility are negatively impacted by forage diets containing less than about 7.5% crude protein (McCollum and Horn, 1990; Moore and Kunkel, 1995). Secondly, in the work of Vona et al. (1984), all forages were fed unprocessed with no indication of concentrate supplementation. In contrast, the current nonlactating evaluation dataset includes 10 of the 19 experiments where the forage was processed and, in many cases, blended with concentrate feeds and (or) a liquid molasses-based supplement. Because the Vona dataset represents ~23% of the data used to derive Equation B (NRC, 1996), these same factors could contribute to the modest underprediction when Equation B was evaluated.

### Evaluation of Existing Equations for Lactating Beef Cows

**Figure 2.**
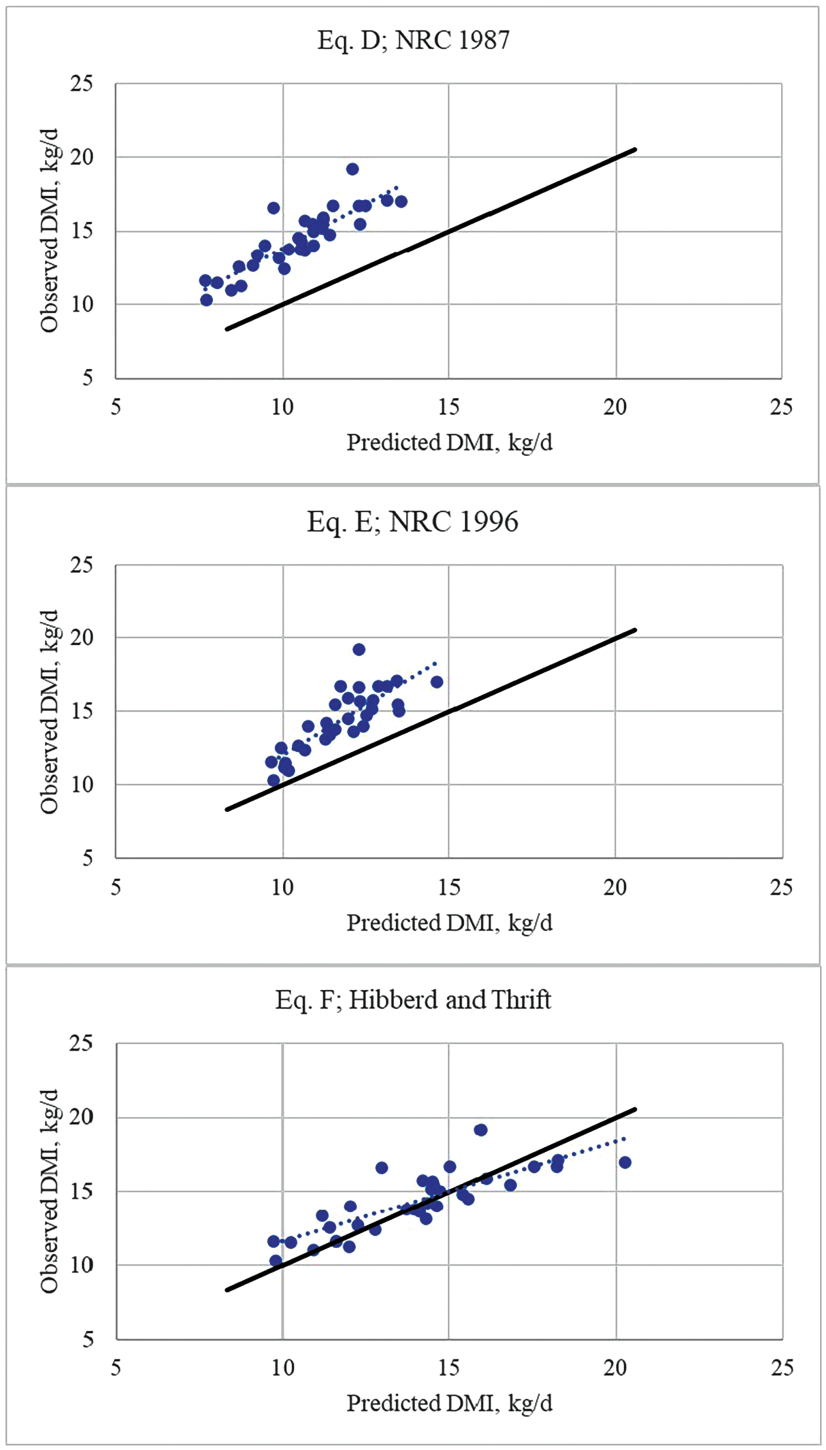
Relationship of observed to predicted feed intake in lactating beef cows using equation D (NRC, 1987), equation E (NRC, 1996), and equation F (Hibberd and Thrift, 1992).

Parameter estimates for regressing observed feed intake against predicted feed intake of lactating cows are provided in Table 3 and a graphical representation is provided in Figure 2. While the predicted values using equation D explained 75% of the variation in observed values and the intercept did not differ from 0, this model was highly inaccurate with a large RMSD due primarily to bias (95%; Tables 3 and 4). As can be seen in Figure 2, equation D grossly underestimated feed intake in all cases. Results from this evaluation reveal the utility of RMSD in evaluating model fit for the purpose of prediction. Regressing observed values against predicted values for equation D give RMSD = 4.1 kg/d average distance from the unity line. For perspective, RMSE = 2.2 kg/d, indicating that the observed values are much closer to the derived least squares regression line (as expected) while remaining distant from the unity line.

Equation E had relatively large RMSD (3.0 kg/d) with a slope significantly different from 1.0 (*P =* 0.001). Like equation D, most of the prediction error was explained by underprediction bias (79%).

As shown in Figure 2, the regression line for equation F crossed the unity line near the center of observed values. As a result, overall prediction error was lower compared to equations D and E. However, both the intercept and slope differed (*P <* 0.01) from 0 and 1, respectively. These results indicate that this equation provided reasonably accurate estimates of feed intake overall, with slight underprediction of feed intake at the lower end of the range in observed values and slight overprediction of feed intake at the higher end of the range in observed values.

Equations A and B are adjusted to a lactating cow basis using a constant to account for increased feed intake relative to milk yield (NRC, 1996; NASEM, 2016). The suggested constant is equal to 0.2 kg for each 1 kg of milk yield. Therefore, assuming the general effects of milk yield, cow weight, and diet energy density are independent, any bias associated with the gestation evaluation results should be reflected in the lactating cow evaluation results because the same coefficients are used. This carryover likely explains some of the dramatic negative bias in equations A and B when applied to lactating cows. The 0.2 kg adjustment was first proposed by ARC (1980) and NRC (1987) using data from dairy cows.

### New Model Development

The best-fit equation to predict daily NEMI included a linear term for diet NE_m_ and an intercept adjustment for STAGE:


$$\begin{array}{lll} NEMI\;= & \;0.224\;\pm \;0.013\;*\;NE_{m}+\;0.0346\;\; & \pm \;0.005\;*\;STAGE\;\;0.142\;\pm \;0.017 \end{array}$$ (2)

where NEMI is the daily NE_m_ intake, kcal/kg SBW^0.75^ and STAGE = 0 for nonlactating and 1 for lactating cows. The interaction term was not significant (*P* = 0.42) and was subsequently removed from the model. Each model component is significant at *P* < 0.01. This model accounted for 79% of the variation (adjusted *R*^2^) in daily NEMI. When both the linear and the quadratic terms for NE_m_ were included in the model, the curve characterizing predicted feed intake (kg/d) produced an illogical concave-shaped curve when plotted against diet NE_m_. This resulted in feed intake (kg/d) predicted to increase at both extremes of the NE_m_ range. Therefore, this model was considered to be overparameterized and not presented.

Predicted daily NEMI was divided by diet NE_m_ concentration to provide estimates of daily feed intake (kg/d). Subsequently, predicted feed intake values were regressed against observed feed intake values resulting in adjusted *r*^2^ = 0.67 and Sy.x = 1.34 kg/d.

The best-fit equation for predicting feed intake (kg/d) directly included linear terms for diet NE_m_ and SBW^0.75^ and an intercept adjustment for STAGE:


$$\begin{array}{l} \begin{array}{lll} DMI=\; & 3.27\pm 0.44*STAGE+9.21\pm 1.18*NE_{m}\; & +0.133\pm 0.017*SBW^{0.75}14.38\pm 2.19\; \end{array} \end{array}$$ (3)

where DMI is the dry matter intake (kg/d), STAGE = 0 for nonlactating and STAGE = 1 for lactating cows, NE_m_ is the diet NE_m_, Mcal/kg, and SBW^0.75^ is the shrunk body weight^0.75^ (kg). The interaction terms were not significant (*P* > 0.27) and were removed from the model. Each predictor variable remaining in the model was significant at *P* < 0.01. The final model (Equation 8) resulted in adjusted *R*^2^ = 0.68 and Sy.x = 1.31.

**Figure 3.**
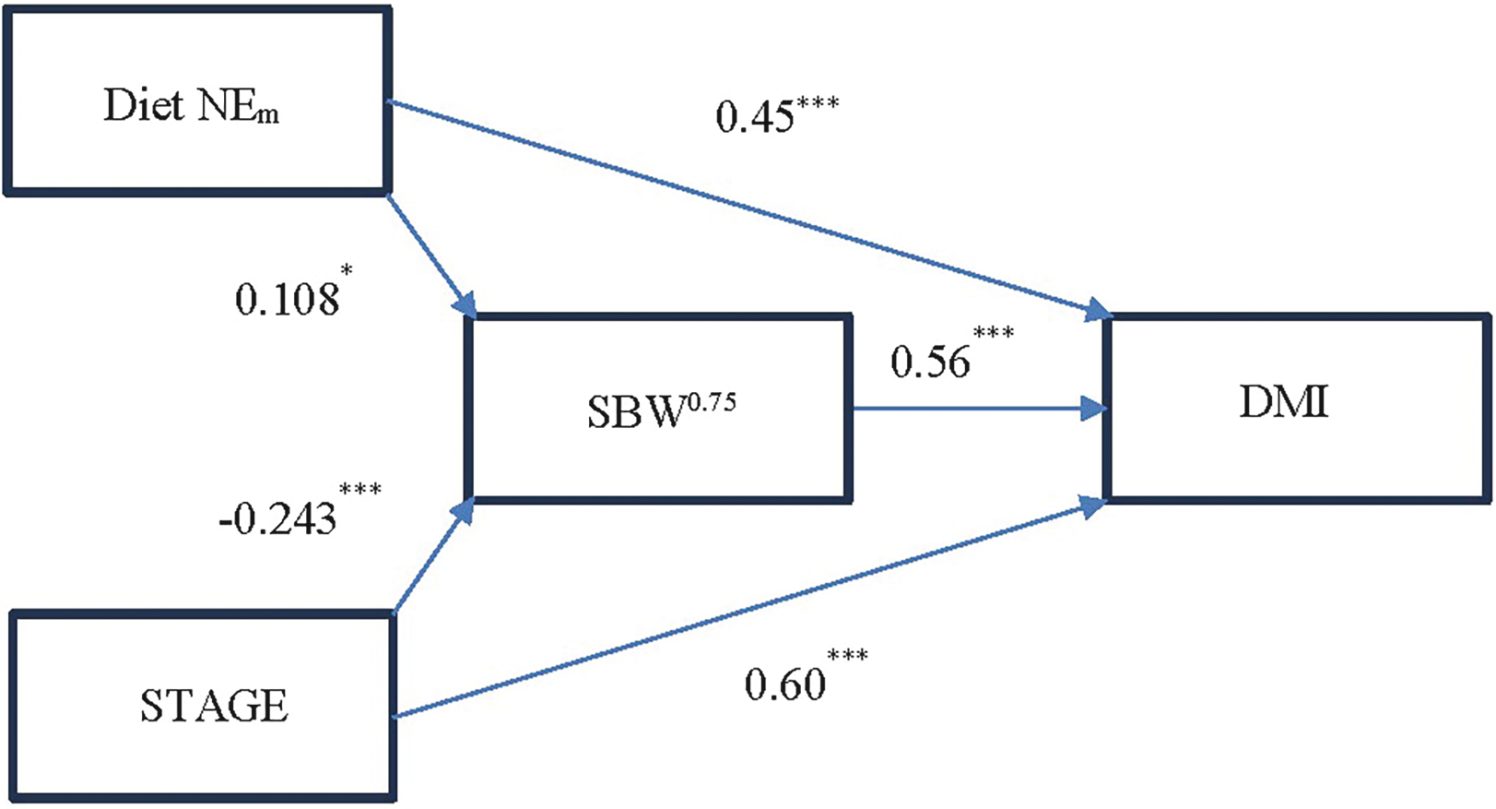
Path analysis to determine the relationship between diet NE_m_ (Mcal/kg), stage of production (STAGE, nonlactating or lactating), shrunk metabolic body weight (SBW^0.75^), and dry matter intake (DMI, kg/d). Standardized path coefficients are shown and significant at *P* < 0.001 (***) and *P* < 0.05 (*).

Path analysis indicated a modest positive indirect effect of diet NE_m_ on DMI through SBW^0.75^ (*P* < 0.05) indicating cattle that consumed energy-dense diets during the study period were heavier and consumed more feed. However, diet NE_m_ also resulted in a strong positive direct effect on DMI (Figure 3; *P* < 0.001) as previously reported (NASEM, 2016). STAGE had a negative indirect effect on DMI (*P* < 0.001) due to lactating cows weighing less than nonlactating cows. However, there was a strong positive direct effect of STAGE, indicating lactation increased DMI (*P* < 0.001). Also as previously reported (NASEM, 2016), SBW^0.75^ had a strong positive direct effect on DMI.

For the 21 treatment means where milk yield was measured by a milking machine, milk yield was 7.3 ± 2.3 kg. In the 11 treatment means available using the weigh-suckle-weigh technique, milk yield was 5.2 ± 1.8 kg with an overall mean of 6.56 ± 2.3 kg. Thus, using the overall mean, the average increase in feed intake per kilogram milk yield is 0.50. Using the yield from machine measurements, feed intake increases, on average, 0.45 kg per kg milk yield. These values are similar to those reported by Johnson et al. (2003; 0.33 and 0.37) and Coleman et al. (2014; 0.55) and substantially greater than the 0.2 kg increase in feed intake per kilogram milk yield recommended by NASEM (2016).

The adjustment for lactation in equation 3 (3.27 kg/d) represents a 21% increase in feed intake for a 545-kg lactating cow consuming a diet with 1.3 Mcal NE_m_ with mean milk yield of 6.56 kg/d. This compares to 1.31 kg/d or 10% increase for the same inputs applied in the current Beef Cattle Nutrient Requirements Model (NASEM, 2016). The increased energy and feed intake required for lactation is associated with energy required to produce milk as well as the increased metabolic activity associated with lactation. For example, maintenance energy requirement has been reported to increase by 5% (Wiseman et al., 2019) to 38% (Neville, 1974) and therefore, maintenance energy requirement in the Beef Cattle Nutrient Requirements Model (NASEM, 2016) is increased by 20% during lactation. This represents an increase of about 2.2 Mcal NE_m_ requirement per day during lactation. In an extensive literature review, the NASEM (2016) committee reported average energy concentration in milk was 0.72 Mcal/kg. Therefore, with mean milk yield of 6.56 kg for these experiments, energy required for lactation is about 6.9 Mcal NE_m_/d (6.56 * 0.72 + 2.2). Given a diet with 1.3 Mcal NE_m_/kg, the feed intake adjustment for lactation given in equation 3 results in increased energy intake of about 4.3 Mcal NEm/d (3.27 * 1.3). When cows consume a diet moderate in energy density, the feed intake response to lactation is not adequate to offset increased energy demand; diet NE_m_ concentration must be increased to avoid negative energy balance.

**Figure 4.**
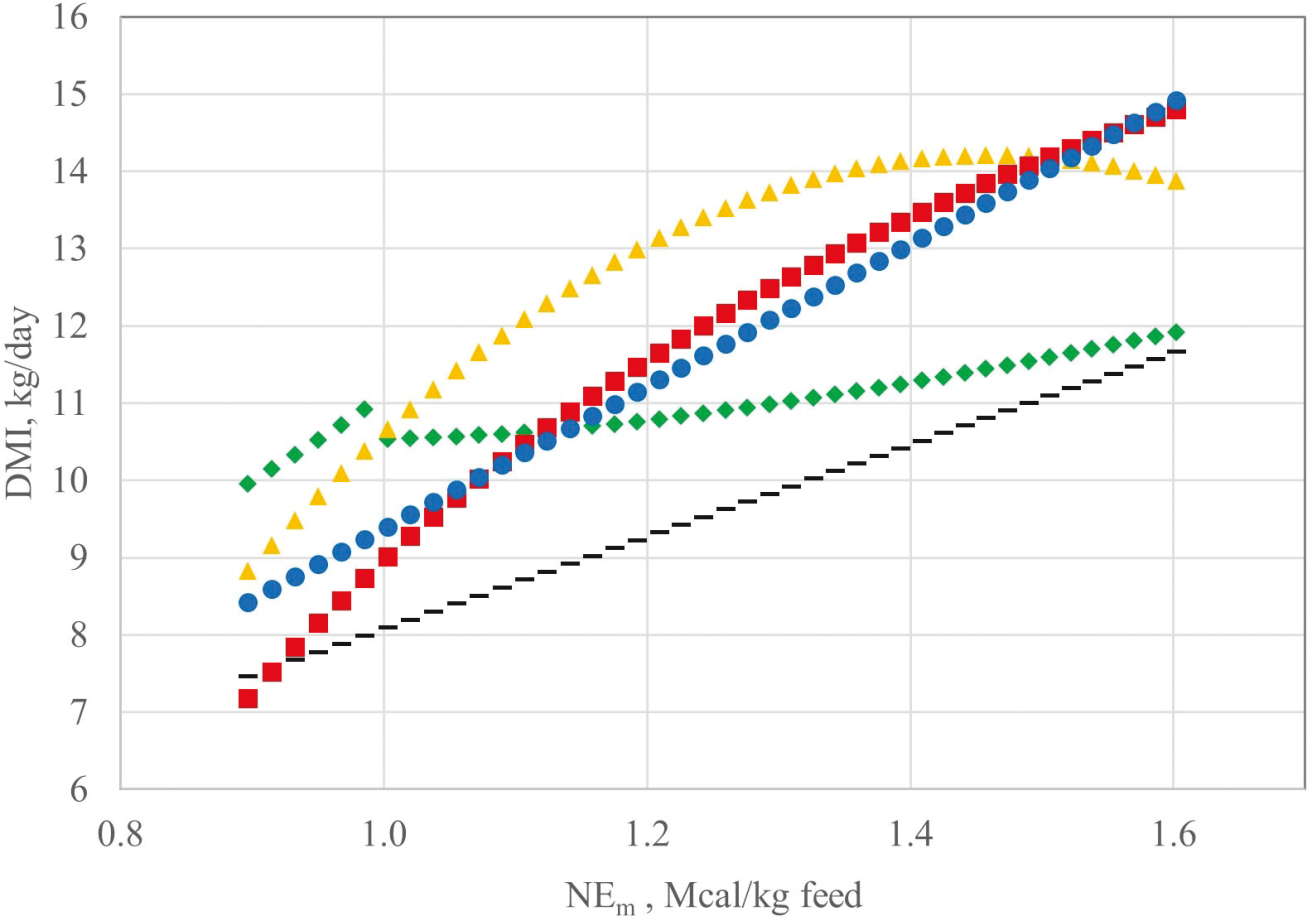
Predicted feed intake response to increasing diet NE_m_ for 545 kg nonlactating beef cows (dashed line, equation A, NRC 1987; diamonds, equation B, NRC 1996; triangles, equation C, Hibberd and Thrift 1992; squares, Equation 2; circles, Equation 3).

**Figure 5.**
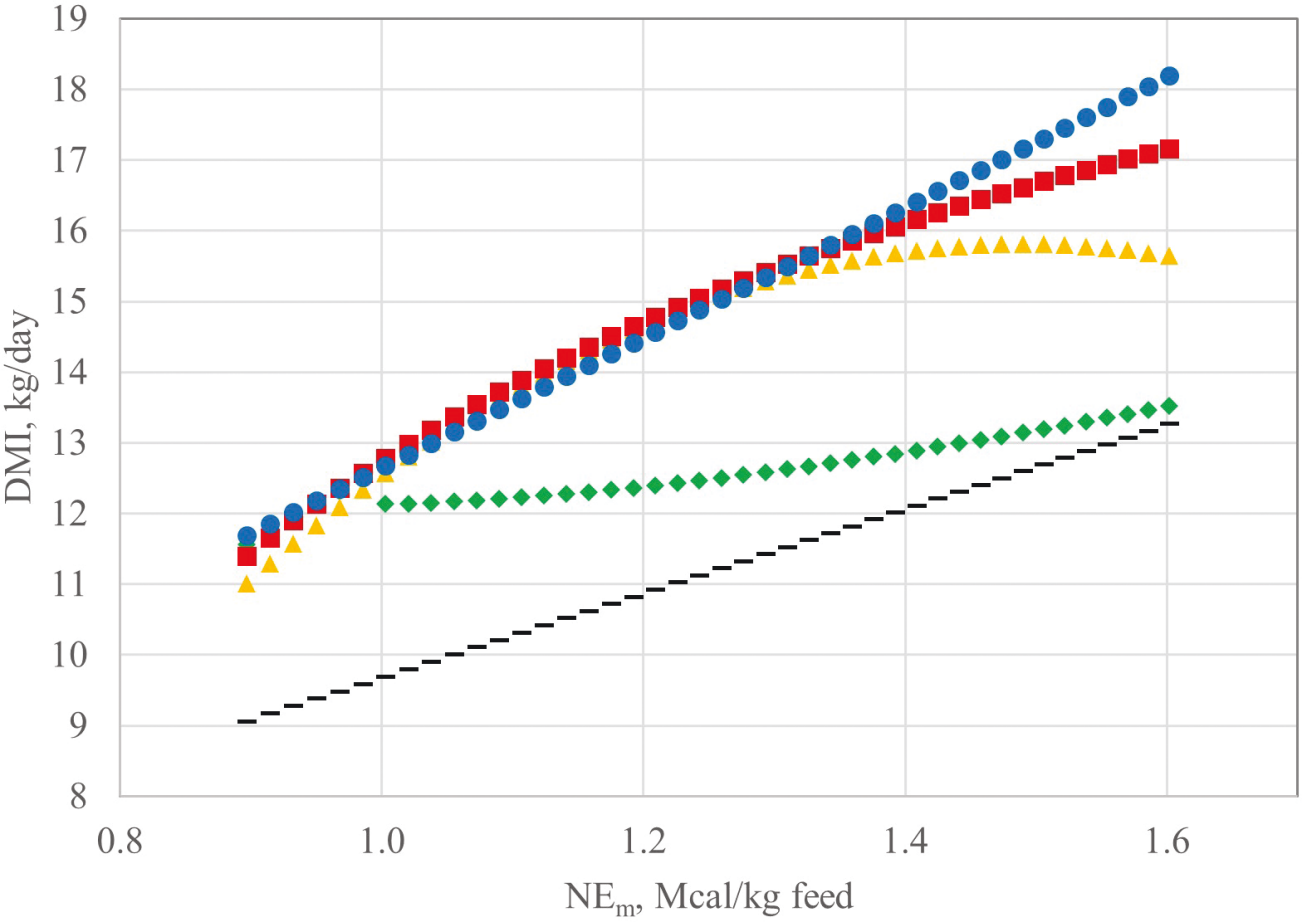
Predicted feed intake for 545 kg beef cows producing 7 kg milk/d (dashed line, equation A, NRC 1987; diamonds, equation B, NRC 1996; triangles, equation C, Hibberd and Thrift 1992; squares, Equation 2; circles, Equation 3).

The relationship between predicted feed intake and diet energy concentration are depicted in Figure 4 for nonlactating cows for equation A (NRC, 1987), equation B (NRC, 1996), equation C (Hibberd and Thrift, 1992), and equations generated from this validation dataset (Equation 2 and 8). Similarly, the same relationships are depicted in Figure 5 for lactating cows using equation D (NRC, 1987), E (NRC, 1996), F (Hibberd and Thrift, 1992), as well as the two new models. The graphical representation reveals the overestimation of feed intake produced by equation C up to about 1.4 Mcal of diet NE_m_. Equations C and F are unique in the stabilizing response to increased diet energy concentration at about 1.4 Mcal NE_m_. The response in cows projected by Equations C and F is similar to the response in growing and finishing beef cattle where feed intake is projected to stabilize between 1.4 and 1.6 Mcal NE_m_ and decline with diet NE_m_ > 1.6 Mcal (NRC, 1984, 1996). This contrasts with equation B, which predicts escalating feed intake when diet energy concentration is beyond about 1.3 Mcal NE_m_. The dataset used to develop equation B has the advantage of a wider range in diet NE_m_ (0.76 to 2.08 Mcal NE_m_) compared to the current evaluation dataset. Clearly, more work is needed to accurately predict feed intake when cows consume high-quality forage or energy-dense mixed diets.

Figure 4 also reveals that equation 2 may underestimate feed intake in nonlactating cows when diet energy concentration is < 1.0 Mcal/kg. For example, with NE_m_ = 0.9 (48 % TDN), equation G predicts nonlactating cows would consume only 1.35% of SBW. However, with diet energy > 1.1 Mcal/kg, DMI predictions are similar for equations G and H (Figure 4).

## Conclusion

Previously published equations (NRC, 1987, 1996) produced reasonable estimates of feed intake in confined, nonlactating, and lactating beef cows only when observed feed intake and (or) diet feed energy concentration was low. These equations dramatically underestimated feed intake when observed feed intake and (or) dietary energy concentration was high. In contrast, the Hibberd and Thrift (1992) equation consistently overestimated feed intake in nonlactating cows, while providing reasonable estimates of feed intake in lactating cows. The new empirical equation (Equation 3) may provide a more accurate estimate of DMI when dietary protein is adequate and within a range of diet energy common to extensive cow/calf production systems. However, validation is necessary for the new equations, particularly over a wide range of diet energy concentrations. Furthermore, characterization of the effects of body composition, grazing activity, forage processing, and milk yield are needed to improve prediction precision and accuracy.

## References

[CIT0001] Agricultural Research Council (ARC). 1980. The nutrient requirements of ruminant livestock. Commonwealth Agricultural Bureau, Farnham Royal, Buckinghamshire, U.K.

[CIT0002] Andresen, C. E., A. W. Wiseman, A. McGee, C. L. Goad, A. P. Foote, R. R. Reuter, and D. L. Lalman. 2020. Maintenance energy requirements and forage intake of purebred vs crossbred beef cows. Trans. Anim. Sci. 4:1–14. doi: 10.1093/tas/txaa008 PMC7201081 32705009

[CIT0003] Anele, U. Y., E. M. Domby, and M. L. Galyean. 2014. Predicting dry matter intake by growing and finishing beef cattle: evaluation of current methods and equation development. J. Anim. Sci. 92:2660–2667. doi: 10.2527/jas.2014-7557 24867938

[CIT0004] Banta, J. P., D. L. Lalman, C. R. Krehbiel, and R. P. Wettemann. 2008. Whole soybean supplementation and cow age class: effects on intake, digestion, performance, and reproduction of beef cows. J. Anim. Sci. 86:1868–1878. doi: 10.2527/jas.2007-0383 18441082

[CIT0005] Black, T. E., K. M. Bischoff, V. R. G. Mercadante, G. H. L. Marquezini, N. DiLorenzo, C. C. Chase, Jr, S. W. Coleman, T. D. Maddock, and G. C. Lamb. 2013. Relationships among performance, residual feed intake, and temperament assessed in growing beef heifers and subsequently as 3-year-old, lactating beef cows. J. Anim. Sci. 91:2254–2263. doi: 10.2527/jas.2012-5242 23463567

[CIT0006] Briggs, E. A., A. L. Holder, M. A. Gross, A. N. Moehlenpah, J. D. Taylor, R. R. Reuter, A. P. Foote, C. L. Goad, and D. L. Lalman. 2022. Retained energy in lactating beef cows; effects on maintenance energy requirements and voluntary feed intake. Trans. Anim. Sci. 6:1–9. doi: 10.1093/tas/txac120 PMC9512099 36172462

[CIT0007] Cassaday, J.C. 2016. Evaluation of intake and feed efficiency measures in beef cattle. PhD Dissertation, University of Illinois. Accessed December 10, 2022. https://core.ac.uk/download/pdf/158315268.pdf

[CIT0008] Coleman, S. W., S. A. Gunter, J. E. Sprinkle, and J. P. Neel. 2014. Beef species symposium: difficulties associated with predicting forage intake by grazing beef cows. J. Anim. Sci. 92:2775–2784. doi: 10.2527/jas.2013-7090 24398834

[CIT0009] Cordova, F. J., J. D. Wallace, and R. D. Pieper. 1978. Forage intake by grazing livestock: a review. J. Range Manag. 31:430–438. doi: 10.2307/3897201

[CIT0010] Fox, D. G., M. C. Barry, R. E. Pitt, D. K. Roseler, and W. C. Stone. 1995. Application of the Cornell Net Carbohydrate and Protein model for cattle consuming forages. J. Anim. Sci. 73:267–277. doi: 10.2527/1995.731267x 7601743

[CIT0011] Freetly, H. C., L. A. Kuehn, R. M. Thallman, and W. M. Snelling. 2020. Heritability and genetic correlations of feed intake, body weight gain, residual gain, and residual feed intake of beef cattle as heifers and cows. J. Anim. Sci. 98:1–6. doi: 10.1093/jas/skz394 PMC6978903 31903482

[CIT0012] Galyean, M. L., and S. A. Gunter. 2016. Predicting forage intake in extensive grazing systems. J. Anim. Sci. 94:26–43. doi: 10.2527/jas2016-0523

[CIT0013] Gauch, H. G., J. G. Hwang, and G. W. Fick. 2003. Model evaluation by comparison of model‐based predictions and measured values. Agron. J. 95:1442–1446. doi: 10.2134/agronj2003.1442

[CIT0014] Gross, M. A., C. Andresen, A. Holder, A. Moehlenpah, C. Goad, H. C. Freetly, P. A. Beck, E. A. Devuyst, and D. L. Lalman. 2020. Predicting dry matter intake in gestating and lactating beef cows. J. Anim. Sci. 98:58. doi: 10.1093/jas/skz397.132. (Abstr.)

[CIT0015] Hibberd, C. A., and T. A. Thrift. 1992. Supplementation of forage-based diets. J. Anim. Sci. 70:181. (Abstr.).

[CIT0016] Holder, A. L., C. E. Andresen, M. A. Gross, A. N. Moehlenpah, and D. L. Lalman. 2020. Voluntary individual intake and performance of mature cows compared to primiparous heifers consuming a low-quality forage. J. Anim. Sci. 98:56. doi: 10.1093/jas/skz397.127. (Abstr.)

[CIT0017] Holder, A. L., M. A. Gross, A. N. Moehlenpah, C. L. Goad, M. Rolf, R. S. Walker, J. K. Rogers, and D. L. Lalman. 2022. Effects of diet on feed intake, weight change, and gas emissions in beef cows. J. Anim. Sci. 100:1–9. doi: 10.1093/jas/skac257 PMC9527298 35952719

[CIT0018] Holder, A. L., J. K. Rogers, R. S. Walker, and D. L. Lalman. 2021. The influence of diet type on ranking for feed intake in angus cows. J. Anim. Sci. 99:32. doi: 10.1093/jas/skab096.057. (Abstr.)

[CIT0019] Holder, A. L., A. Wiseman, A. McGee, C. Andresen, and D. L. Lalman. 2019. The effect of fleshing ability on cow and calf performance. J. Anim. Sci. 97:22. doi: 10.1093/jas/skz053.049. (Abstr.)

[CIT0020] Holecheck, J. L., M. Vavra, and R. D. Pieper. 1982. Methods for determining the nutritive quality of range ruminant diets: a Review. J. Anim. Sci. 54:363–376. doi: 10.2527/jas1982.542363x

[CIT0021] Jardstedt, M., A. Hessle, P. Nørgaard, L. Frendberg, and E. Nadeau. 2018. Intake and feed utilization in two breeds of pregnant beef cows fed forages with high-fiber concentrations. J. Anim. Sci. 96:3398–3411. doi: 10.1093/jas/sky199 29790935 PMC6095262

[CIT0022] Johnson, C. R., D. L. Lalman, M. A. Brown, L. A. Appeddu, D. S. Buchanan, and R. P. Wettemann. 2003. Influence of milk production potential on forage dry matter intake by multiparous and primiparous Brangus females. J. Anim. Sci. 81:1837–1846. doi: 10.2527/2003.8171837x 12854822

[CIT0023] Kobayashi, K., and M. U. Salam. 2000. Comparing simulated and measured values using mean squared deviation and its components. Agron. J. 92:345–352. doi: 10.1007/s100870050043

[CIT0024] Lalman, D. L., C. E. Andresen, A. L. Holder, R. R. Reuter, and A. P. Foote. 2019. Application of the California Net Energy System to grazed forage: feed values and requirements. Transl. Anim. Sci. 3:962–968. doi: 10.1093/tas/txz034 32704860 PMC7200905

[CIT0025] Langlands, J. P. 1974. Studies on the nutritive value of the diet selected by grazing sheep VII A note on hand-plucking as a technique for estimating dietary composition. Anim. Prod. 19:219–252. doi: 10.1017/S0003356100022807

[CIT0026] Martin, P., S. Taussat, A. Vinet, D. Krauss, D. Maupetit, and G. Renand. 2019. Genetic parameters and genome-wide association study regarding feed efficiency and slaughter traits in Charolais cows. J. Anim. Sci. 97:3684–3698. doi: 10.1093/jas/skz240 31436836 PMC6735829

[CIT0027] McCollum, F. T., III. G. W. Horn . 1990. Protein supplementation of grazing livestock: a review. Prof. Anim. Sci. 6:1–16. doi: 10.15232/S1080-7446(15)32251-8

[CIT0028] Moehlenpah, A. N., L. P. S. Ribeiro, R. Puchala, A. L. Goetsch, P. Beck, A. Pezeshki, M. A. Gross, A. L. Holder, and D. L. Lalman. 2021. Water and forage intake, diet digestibility, and blood parameters of beef cows and heifers consuming water with varying concentrations of total dissolved salts. J. Anim. Sci. 99:1–10. doi: 10.1093/jas/skab282 PMC8557798 34618893

[CIT0029] Moore, J.E., and W.E. Kunkle. 1995. Improving forage supplementation programs for beef cattle. : Proc. 6th Annu. Rumin. Nutr. Symp., Gainesville, FL; p. 65–74.

[CIT0030] Moore, M. F., E. A. Briggs, D. L. Lalman, and A. Holder. 2022. Ranking mature beef cows for residual intake using an unprocessed grass hay diet and its relationship to greenhouse gas exchange. J. Anim. Sci. 100:35. doi: 10.1093/jas/skac028.067

[CIT0031] Mourer, G. L. 2012. Effects of cow mature size on intake, calf weight and milk yield in a spring-calving commercial cow/calf operation (master’s thesis) Oklahoma State University, Stillwater, Oklahoma, U.S.

[CIT0032] Mourer, G. L., C. P. McMurphy, A. J. Sexten, C. D. Dobbs, S. K. Linneen, J. D. Sparks, and D. L. Lalman. 2012. Effects of mature size on intake, calf weight, and milk yield in a spring-calving commercial cow/calf operation. J. Anim. Sci. 90 (e-Suppl. 2):5.

[CIT0033] National Academies of Sciences, Engineering, and Medicine. 2016. Nutrient requirements of beef cattle. 8th rev. ed. Washington, DC: The National Academies Press; doi: 10.17226/19014

[CIT0034] National Research Council (NRC). 1984. Nutrient requirements of beef cattle. Washington, DC: The National Academies Press; doi: 10.17226/19398

[CIT0035] National Research Council (NRC). 1987. Predicting feed intake of food-producing animals. Washington, DC: The National Academies Press, National Academy of Science; doi: 10.17226/950

[CIT0036] National Research Council (NRC). 1996. Nutrient requirements of beef cattle, 7th Revised Edition: Update 1996. Washington, DC: The National Academies Press; doi: 10.17226/9791

[CIT0038] Neal, H. D., C. Thomas, and J. M. Cobby. 1984. The grasslands research institute Comparison of equations for predicting voluntary intake. J. Agric. Sci., Camb. 103:1–10. doi: 10.1017/S0021859600043264

[CIT0039] Neville, W. E. Jr . 1974. Comparison of energy requirements of nonlactating and lactating Hereford cows and estimates of energetic efficiency of milk production. J. Anim. Sci. 38:681–686. doi: 10.2527/jas1974.383681x 4819556

[CIT0040] Parsons, C. T., J. M. Dafoe, S. A. Wyffels, T. DelCurto, and D. L. Boss. 2021. Influence of residual feed intake and cow age on dry matter intake post-weaning and peak lactation of black Angus cows. Animals. 11:1822–1829. doi: 10.3390/ani11061822 34207267 PMC8234949

[CIT0042] Piñeiro, G., S. Perelman, J. P. Guerschman, and J. M. Paruelo. 2008. How to evaluate models: observed vs predicted or predicted vs observed? Ecol. Model. 216:316–322. doi: 10.1016/j.ecolmodel.2008.05.006

[CIT0044] Sexten, A. J., M. F. Moore, C. P. McMurphy, G. L. Mourer, S. K. Linneen, M. A. Brown, C. J. Richards, and D. L. Lalman. 2021. Effects of bale feeder design on hay waste, intake, and apparent diet digestibility in gestating beef cows. Trans. Anim. Sci. 5:1–10. doi: 10.1093/tas/txab104 PMC8281098 34278238

[CIT0045] Vona, L. C., G. A. Jung, R. L. Reid, and W. C. Sharp. 1984. Nutritive value of warm-season grass hays for beef cattle and sheep: digestibility, intake and mineral utilization. J. Anim. Sci. 59:1582–1593. doi: 10.2527/jas1984.5961582x

[CIT0046] Wagner, J. J., K. S. Lusby, J. W. Oltjen, J. Rakestraw, R. P. Wettemann, and L. E. Walters. 1988. Carcass composition in mature Hereford cows: estimation and effect on daily metabolizable energy requirement during winter. J. Anim. Sci. 66:603–612. doi: 10.2527/jas1988.663603x 3378920

[CIT0047] Walker, R. S., R. M. Martin, G. T. Gentry, and L. R. Gentry. 2015. Impact of cow size on dry matter intake, residual feed intake, metabolic response, and cow performance. J. Anim. Sci. 93:672–684. doi: 10.2527/jas.2014-7702 25548208

[CIT0048] Warren, C. 2017. Factors affecting the determination of residual feed intake in beef cows fed a common diet in mid to late gestation. Thesis, The University of Guelph. Accessed December 12, 2022. https://atrium.lib.uoguelph.ca/xmlui/handle/10214/10445

[CIT0049] Williams, A. R., C. T. Parsons, J. M. Dafoe, D. L. Boss, J. G. Bowman, and T. DelCurto. 2018. The influence of beef cow weaning weight ratio and cow size on feed intake behavior, milk production, and milk composition. Transl. Anim. Sci. 2:79–83. doi: 10.1093/tas/txy044 PMC7200941 32704741

[CIT0050] Winterholler, S. J., D. L. Lalman, M. D. Hudson, and C. L. Goad. 2009. Supplemental energy and extruded-expelled cottonseed meal as a supplemental protein source for beef cows consuming low-quality forage. J. Anim. Sci. 87:3003–3012. doi: 10.2527/jas.2008-1605 19542511

[CIT0051] Wiseman, A., M. Redden, A. McGee, C. Spencer, R. Reuter, G. Horn, and D. Lalman. 2019. Effects of timing of weaning on energy utilization in primiparous beef cows and post-weaning performance of their progeny. J. Anim. Sci. 97:1198–1211. doi: 10.1093/jas/skz019 30668783 PMC6396231

